# Correction: Hepaticojejunostomy bile leakage and aberrant biliary anatomy during robotic pancreatoduodenectomy: intraoperative detection and management

**DOI:** 10.1007/s00464-026-13400-5

**Published:** 2026-09-17

**Authors:** Oskar Swartling, Julia E. Menso, Freek Daams, Sebastiaan Festen, Martin C. Boonstra, Babs Zonderhuis, Joris Erdmann, Rutger-Jan Swijnenburg, Olivier R. Busch, Marc G. Besselink

**Affiliations:** 1 Department of Upper Abdominal Diseases, Karolinska University Hospital, Stockholm, Sweden; 2 Division of Surgery and Oncology, Department of Clinical Science, Interventions and Technology, Karolinska Institutet, Stockholm, Sweden; 3 Amsterdam UMC, University of Amsterdam, Department of Surgery, Amsterdam, The Netherlands; 4 Cancer Center Amsterdam, Amsterdam, The Netherlands; 5 Amsterdam UMC, Vrije Universiteit, Department of Surgery, Amsterdam, The Netherlands; 6 Department of Surgery, OLVG, Amsterdam, The Netherlands

**Correction to: Surgical Endoscopy** **https://doi.org/10.1007/s00464-026-13067-y**

The original online version of this article was revised to add “for HPB-Amsterdam” to the end of the list of contributing authors to indicate collaboration.

The original article has been corrected.

